# Quantum JIDOKA. Integration of Quantum Simulation on a CNC Machine for In–Process Control Visualization

**DOI:** 10.3390/s21155031

**Published:** 2021-07-24

**Authors:** Javier Villalba-Diez, Miguel Gutierrez, Mercedes Grijalvo Martín, Tomas Sterkenburgh, Juan Carlos Losada, Rosa María Benito

**Affiliations:** 1 Hochschule Heilbronn, Fakultät Management und Vertrieb, Campus Schwäbisch Hall, 74523 Schwäbisch Hall, Germany; 2 Complex Systems Group, Universidad Politécnica de Madrid, Av. Puerta de Hierro 2, 28040 Madrid, Spain; juancarlos.losada@upm.es (J.C.L.); rosamaria.benito@upm.es (R.M.B.); 3 Department of Organizational Engineering, Escuela Técnica Superior de Ingenieros Industriales, Universidad Politécnica de Madrid, 28006 Madrid, Spain; miguel.gutierrez@upm.es (M.G.); mercedes.grijalvo@upm.es (M.G.M.); 4 Independent Researcher, Niesmannshof 54, 46535 Dinslaken, Germany; sterkenburgh@t-online.de

**Keywords:** quantum simulation, JIDOKA, industry 4.0, shopfloor management

## Abstract

With the advent of the Industry 4.0 paradigm, the possibilities of controlling manufacturing processes through the information provided by a network of sensors connected to work centers have expanded. Real-time monitoring of each parameter makes it possible to determine whether the values yielded by the corresponding sensor are in their normal operating range. In the interplay of the multitude of parameters, deterministic analysis quickly becomes intractable and one enters the realm of “uncertain knowledge”. Bayesian decision networks are a recognized tool to control the effects of conditional probabilities in such systems. However, determining whether a manufacturing process is out of range requires significant computation time for a decision network, thus delaying the triggering of a malfunction alarm. From its origins, JIDOKA was conceived as a means to provide mechanisms to facilitate real-time identification of malfunctions in any step of the process, so that the production line could be stopped, the cause of the disruption identified for resolution, and ultimately the number of defective parts minimized. Our hypothesis is that we can model the internal sensor network of a computer numerical control (CNC) machine with quantum simulations that show better performance than classical models based on decision networks. We show a successful test of our hypothesis by implementing a quantum digital twin that allows for the integration of quantum computing and Industry 4.0. This quantum digital twin simulates the intricate sensor network within a machine and permits, due to its high computational performance, to apply JIDOKA in real time within manufacturing processes.

## 1. Introduction

Driven by an unprecedented level of transparency based on the global availability of information, companies are facing extremely competitive global markets in which customers’ expectations have risen to demand very high quality standards at a low price and ever-increasing speeds [1]. Adding to this, requests for customised products are growing as to become the pattern in certain industries [2]. Manufacturing industry is relying on technology to face this challenging environment, with Industry 4.0 emerging as a paradigm that can provide solutions to keep track of the markets [3,4].

Kagermann [5] places cyber-physical systems (CPS) as the key driver to trigger the Industry 4.0 paradigm, paralleling its role with the one played, respectively, by steam machines, mass production lines, and integrated circuits in the previous three industrial revolutions. CPS can be defined as “systems of collaborating computational entities which are in intensive connection with the surrounding physical world and its on-going processes, providing and using, at the same time, data-accessing and data-processing services available on the Internet” [6]. As stated by Lee, Bagheri, and Kao [7] “a CPS consists of two main functional components: (1) the advanced connectivity that ensures real-time data acquisition from the physical world and information feedback from the cyber space; and (2) intelligent data management, analytic and computational capability that constructs the cyber space”. Thus, CPS lead to a decentralised control system characteristic of the Industry 4.0, in which machines show great autonomy, share information with other machines, and handle large amounts of data.

For Industry 4.0 to be effective, interdisciplinary knowledge from engineering, computer science, business, and various other academic disciplines is crucial [8]. Lean Management stands out as a consolidated managerial paradigm that tries to tackle the previously mentioned challenges, with a large history since it was grounded in the 1940s by Toyota production managers [9,10,11,12]. The main goal of both Industry 4.0 and lean management is to find ways to deal with an ever increasing complexity in the manufacturing industry that nowadays partially stems from digitisation, as well as mass customisation. Although Industry 4.0 puts the focus on the technological elements, lean methods seek to find ways to reduce the complexity by designing clear and controllable processes that minimise non value-adding activities throughout the value chain [13]. To achieve this goal, a wide range of tools, initially conceived under the umbrella of the Toyota just-in-time (JIT) system, such as synchronised production, Kanban, single minute exchange of die (SMED), cross-functional work force [14], and others, that evolved to gain its own field of development as total productive maintenance (TPM) or total quality management (TQM) [15], have been developed and put into practice in manufacturing environments. These techniques have shown significant positive effects in different industries and even synergistic benefits through their combined implementation [16,17]. Combining the methodologies from Industry 4.0 and lean manufacturing has been an increasingly popular research topic, resulting in the so-called Lean 4.0 [18]. There are mixed opinions regarding whether lean management is needed to enable Industry 4.0 or Industry 4.0 advance lean management [19]. Yet, one of the key ideas about Industry 4.0 is the integration of varied technologies due to the limited effect of focusing on a single technology [20]. In fact, several of the many lean tools have been examined in the context of Industry 4.0 [21,22,23], leading to the conclusion that a leaner production is easier to integrate into an Industry 4.0 context and, in many cases, it is possible to combine the ideas and techniques from both frameworks [24,25,26,27,28]. Moreover, it seems to be a necessary evolutionary step for further raising the level of operational excellence (i.e., to coordinate actions, to optimise resource efficiency, to improve work safety, to decrease in cost) [18]. In this sense, one successful Lean 4.0 approach is the integration of JIDOKA(自働化) into the Industry 4.0 framework, which has shown great potential [29,30,31] and motivates this work.

JIDOKA(自働化) is currently considered a lean tool that “enables machines to work harmoniously with their human operators and features intelligent capabilities by automatically stopping a process by man or machine, in the event of an abnormally, a problem, such as equipment malfunction, quality issues, or late work” [30,32]. It was introduced by Ohno [14] as one of the two pillars of the Toyota production system, alongside with the JIT, needed to accomplish the elimination of waste. The Japanese term was translated from the coined term “autonomation” or “automation with human touch” and its origins traced back to the invention by Sakichi Toyoda (1867–1930) of a looming machine that would automatically stop as soon as a thread in the machine teared [14]. The core idea behind JIDOKA is to provide “intelligence” to machines with built-in automatic checking systems that would automatically stop to prevent any defective part passing to the next step in the value stream. In case of an abnormal situation, the intervention of an operator that can be in charge of monitoring several processes is necessary. Moreover, to eliminate any source of waste, JIDOKA aims at preventing this mistake from happening, again, reinforcing a culture of continuous learning and improvement. Thus, it is essential to identify the defective part as soon as possible, trigger a signal to stop the work center, and even the production process if necessary, to determine what the root cause for the production of the defective item has been, but also achieve an effective human–machine interaction. For many years, JIDOKA principles were primarily built on mechanical tools and devices with electronic components playing an increasing role. Romero et al. [30] describe three prior generations of JIDOKA systems: mechanical gadgets that avoid mistakes (POKA–YOKE (ポカヨケ)), visual and audio alarms (ANDON (行灯)), sensor-based fault diagnosis (JIDOKA(自働化) rules). The immense possibilities brought up by Industry 4.0, through digitisation and wide availability of low-cost sensors, open the way to utilise these large amounts of data and be able to act on a variety of input variables and handle complex processes and lead to a new generation JIDOKA 4.0 [30]. Hence, it becomes an example of Industry 4.0 enabling the further integration of lean tools [31]. The impact of Industry 4.0 in the evolution of JIDOKA 4.0 is described in what follows.

As aforementioned, the main core of JIDOKA (自働化), which is the stopping of the machine, can only be one of the first steps as still no further production should take place until the operator has found the mistake. This has been one of the main reasons why many companies, outside Japan, were hesitant at first about implementing JIDOKA(自働化) [14]. Even the use of Andon-systems, which notify the correct person of the shutdown can only reduce but not eliminate those negative effects. The next step is to make the data available to the operator, in order to assist the search for the source of the defective machine. Industry 4.0 can act as a key enabler [33]. Ma, Wang, and Zhao [29] show a successful implementation of a CPS-based JIDOKA (自働化) system. In addition to its technological side, CPS offer multimodal interfaces for more effective human–machine collaboration [34]. It is part of the Industry 4.0 networking concept, production and people as entities, participating in value creation [35]. Another step further consists in directly pointing out the possible source of the problem. An ideal goal is the design of a self-regulating machine, which is able to adapt to different circumstances and prevent the need of stopping the machine. Although there are some limitations to identify the cause of defective parts in all cases, it should be possible to be able to at least better predict whether a part is likely to break. Deuse et al. [31] outline a generic proposal of a system aiming at this objective. This approach relates to maintenance systems where sensors are installed in different parts of the machine to monitor its operation in real time [36]. The data that these sensors send, consequently structured and appropriately processed, allows predicting the failure of the monitored spare parts of the machine. On the other hand, due to the availability of low-cost sensors and suitable mechanisms of sensor integration—e.g., by powerful mobile networks—the amount of available data is drastically increasing. With the benefits of availability comes the effort of analysis and handling of big data. In addition, the human interface needs an appropriate reduction in complexity to enable an effective JIDOKA (自働化) process [32]. Although the target range and probability distributions for the corresponding measured values can be specified for each sensor, the evaluation of sensor data due to networked conditional probabilities creates a high degree of complexity, which quickly overtaxes even experienced employees in the evaluation. Incorrect evaluations and time delays are often the result. In this respect, supporting a system that automatically performs the task of pre-assessing the overall situation for the human as a result of the interaction of the individual measured values can be of considerable benefit. Decision networks, which quickly and efficiently calculate the conditional probabilities of the interconnection of random variables, can be an effective tool to this aim [37]. However, even this approach faces limitations due to the computational power required and leads to delays in feedback to the worker [38]. Quantum simulations shows relevant advantages to accelerate this feedback significantly [39,40]. Hence the interest in studying the possibilities of quantum computing to implement the described JIDOKA (自働化) methodology more efficiently and responsively.

In sum, the growing possibilities of combining lean manufacturing and Industry 4.0 and their associated benefits have been posed by many studies [21,22,23,24,26,28], whereas the rapid evolution of technological advancements opens new fields of applications. Previous works have shown that JIDOKA(自働化) benefits from some Industry 4.0 technologies such as CPS, connectivity, and operator wearable systems [26,29,33], and recently to improve process monitoring in order to predict quality defects [31]. An important aspect in the implementation of JIDOKA (自働化) is the operator, as the system only works through the cooperation of both [30]. Efficient Bayesian network computing algorithms can help firms to adopt JIDOKA (自働化) or “automation with human touch” approach, not only improving the efficiency of the manufacturing process but also making possible a human–machine cooperation system. In this context, we propose the integration of quantum computing and JIDOKA(自働化) to simulate the intricate monitoring sensor network within a machine. This quantum simulation of the machine sensor network is a quantum digital twin.

This quantum digital twin will allow—due to the speed that quantum computing offers—to apply real-time JIDOKA (自働化) in the manufacturing processes. Since this work is oriented to Industry 4.0 users and we want to give it an eminently experimental and practice-oriented character, we prefer to focus on the core of the problem and thus spare the reader from lengthy theoretical explanations on the design of quantum simulations. In references [41,42,43,44], interested readers can find papers in which the authors show both the theoretical and practical principles of how to transform decision networks with conditional probabilities into their counterpart quantum circuits.

The remainder of the paper is organised as follows: in Section 2 the case study chosen to test our hypothesis is introduced: we describe the structure and interaction of the overall system with our quantum digital twin and establish the scope in Section 2.1. In Section 2.2 the specific hardware and software are presented, followed by the description of the data collection components of the setup in Section 2.3. Results of our study are summarised in Section 3. Finally, further aspects are discussed in Section 4, where we also draw the main conclusions, limitations, and future applications of our work.

## 2. Case Study Quantum JIDOKA

**Figure 1:**
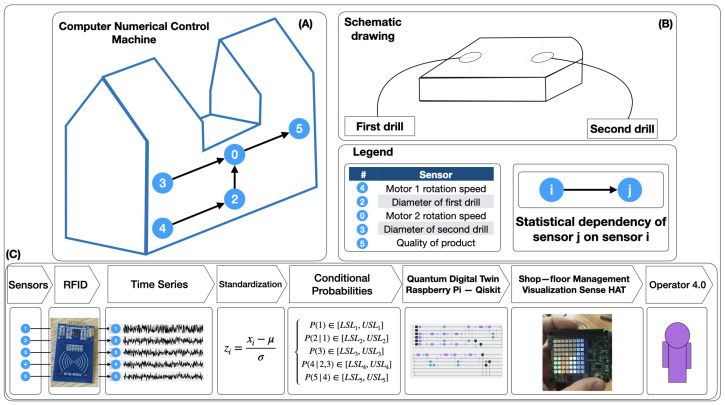
Scheme of our Quantum JIDOKA case study: the digital quantum twin to provide the 4.0 operator with a shopfloor management tool that allows him to visualize the status of the machine in real time. (**A**) CNN-machine and schematic sensor network. (**B**) Schematic product drawing. (**C**) Quantum (自働化) creation process.

Our initial working hypothesis is that we can model the internal sensor network of a machine with quantum simulations that show better performance than classical models based on decision networks. To quantitatively test this hypothesis, as a first step to evaluate the effect of the integration of quantum simulations in I4.0 environments, a case study is used. In this case study, we are going to generate a digital quantum twin to provide the 4.0 operator with a shopfloor management tool that allows him to visualize the status of the machine in real time, as shown schematically in Figure 1.

We integrated the proposed solution in a factory and tested it for 12 weeks before submitting the work. However, for privacy reasons imposed by the process owner, the data related to the process in question cannot be disclosed. For this reason a dummy dataset is presented to show the functionality of the solution, as well as to ensure reproducibility to interested scholars or industrialists. The machine in question consists of 5 sensors that measure the rotational speed of two motors that, in turn, drive two drills that make two holes, whose relative position determines the quality of the final product. As argued by Byrd and Turner [45], a single case study can be seen as the only possible building block in the process of developing the validity and reliability of the proposed hypothesis. Following the recommendations of Eisenhardt [46], a clear case study road-map is followed for each one of them. This road-map has several phases: (1) scope establishment, (2) specification of hardware and software, (3) data collection, and (4) quantum digital twin.

### 2.1. Scope Establishment

The use of quantum computers in the implementation of larger decision networks and their use in a JIDOKA (自働化) environment should open up the possibility of performing high-performance analysis in larger sensor networks.

The objective of this case study is to generate a low-cost quantum digital twin that represents the statistical dependencies between five sensors positioned on a computer numerical control processing machine that measure parameters relevant to the quality of the manufactured product. For this purpose, we will install a quantum circuit in a low-cost component, which simulates the statistical dependencies derived from the value-creation process inside the machine. This component will receive data from the machine through a radio frequency identification (RFID) device and will compute the state of the machine in real time, generating a visualization that will allow the process owner to understand it. This interface will resemble a conditioning monitoring and will allow for a preventive operational lean shopfloor management.

The implementation of a quantum computer simulation on a Turing machine is certainly accompanied by limitations, which are reflected in the performance and low number of manageable measured values. Nevertheless, the setup chosen here allows a feasibility study and can serve as a basis for later scaling when used in a real system environment and with the use of adequate quantum computers.

### 2.2. Specification of Hardware and Software

The proposed equipment has a low-cost standard configuration and the components can be easily available on the market. This design was consciously chosen for two reasons: on the one hand reliability is increased as the compounds are well proven, on the other hand an adequate stock of spare parts can be stored to ensure the continuous operation of the process at a very low cost.

#### 2.2.1. Hardware

**Figure 2:**
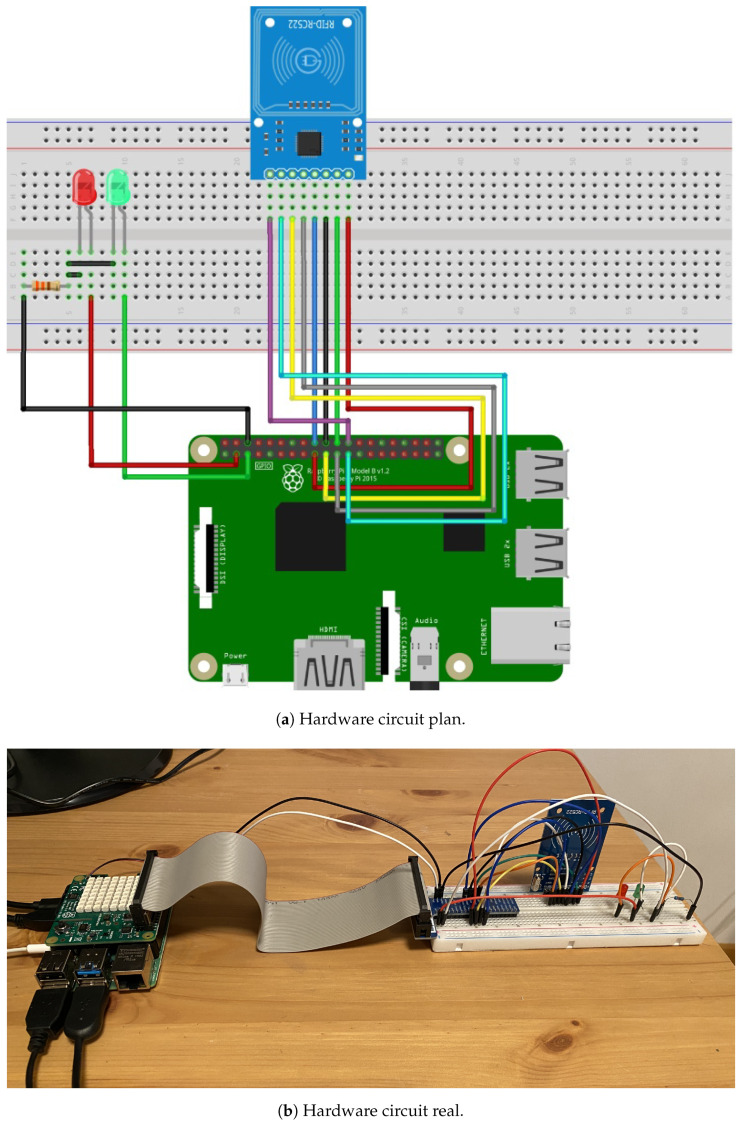
RFID LEDs Raspberry Pi Visualization Hat-Hardware.

The necessary hardware needed to build the smart IoT sensor prototype is shown in Figure 2:

- Raspberry Pi 4.0. The Raspberry Pi 4.0 is a high-performance 64-bit quad-core processor, up to 8 GB of RAM, dual-band 2.4/5.0 GHz wireless local area network (LAN), Bluetooth 5.0, Gigabit Ethernet, USB 3.0, and a sense HAT add-on which is attached on top of the Raspberry Pi via the 40 general-purpose input or output pins (which provide the data and power interface). It has several sensors and an 8 × 8 RGB (Red–Green–Blue) LED matrix display that can be used to visualize sensor states for multiple applications [47,48,49,50]. In the proposed design, the critical component, because of its value and relative complexity is the Raspberry Pi CPU. In the factory in question there are about two hundred CPUs of this type, and the annual failure rate, including human-caused failures, is 1%. This is acceptable and within standard maintenance parameters.
- RC522 RFID module. The RC522 RFID is a 13.56 MHz RFID module that is based on the MFRC522 controller from NXP semiconductors. Its operating voltage lies between 2.5 V to 3.3 V. It allows for serial peripheral interface (SPI), inter-integrated circuit (I${}^{2}$C) and universal asynchronous receiver and transmitter (UART) communication protocols. Its maximum data rate is 10 Mbps with a read range of 5 cm and a current consumption of 13 to 26 mA. These characteristics are optimal for a number of industrial and educational applications [51,52].
- Set of cables, light-emitting diodes (LEDs), resistors, and test plates. An LED is a two-lead semiconductor light source, which emits light when activated. The LEDs used present a forward current of 30 mA and a forward voltage range between 1.8 V and 2.4 V. A resistor is a passive electronic component that offers a specific amount of electrical resistance to the flow of current when connected in a circuit. The resistors used in this project are standard 1 kΩ.

The Raspberry Pi is connected to the RFID card and the two red and green LEDs show the status of the connection. The electrical resistance allows for a proper functioning of the elements.

#### 2.2.2. Software

The quantum digital twin circuits presented in Section 2.4 were simulated on *qiskit* tool, a Python-based [53] quantum computing platform developed by IBM [54], and the code and additional results can be accessed in this Open Access Repository: https://osf.io/24jrm/?view_only=a10d2e001e114807854b994616f8d4cf. We will analyze the data obtained in Section 2.3, evaluate these results in Section 3, and discuss the results obtained in Section 4.

### 2.3. Data Collection

**Figure 3:**
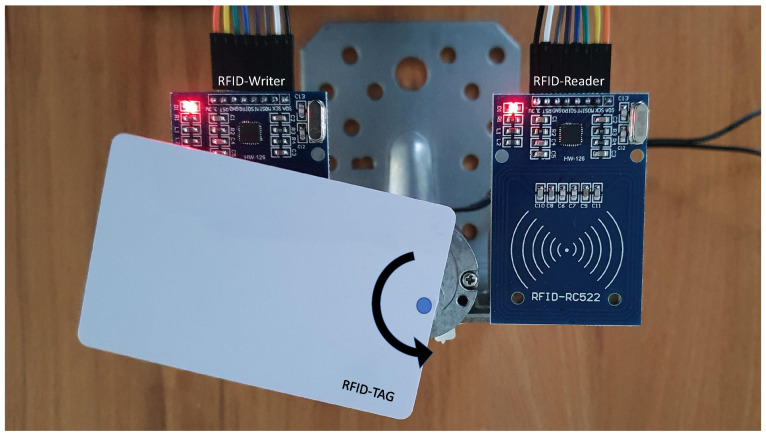
RFID data collection.

The data input is realised by means of a radio frequency identification (RFID) module, connected to the serial peripheral interface bus (SPI) of the Raspberry Pi as presented in Figure 2b. This module acts as RFID-reader. To simulate a more realistic industrial process, we set up a data transfer by RFID consisting of the following components:

- An RFID-writer, connected to the sensors of the computer numerical control machine in Figure 1;
- The RFID-reader as described in Figure 2;
- A small machine that is physically rotating an RFID-card around a motor-driven axis, as shown in Figure 3, and by this transferring datasets from the writer to the reader.

One transfer process consists of one revolution of the RFID-card around the axis. Per revolution, one dataset is transferred. Each dataset consists of one measured value per sensor, as well as a time stamp and a quality assessment in the form of “ok” or “not ok” information. The process starts with the RFID-card in reach for the RFID-writer. Here, one dataset from the computer numerical control machine is written to the RFID-Tag. After that, the card is rotating around the motor axis. With a 180 degree rotation, the card comes into reach of the RFID reader. Here, the dataset is read from the RFID card, split into its components, entered into one list per sensor on the Raspberry Pi, and thereby made available on the digital twin.

### 2.4. Quantum Digital Twin

**Figure 4:**
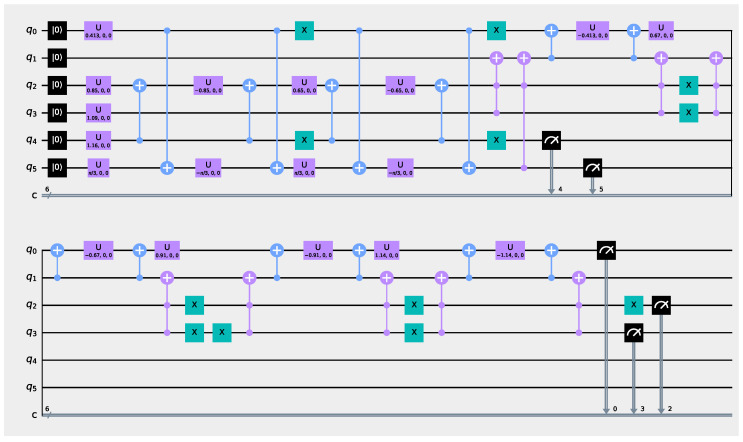
Quantum digital twin for the sensor network shown in Figure 1.

The quantum digital twin circuit that resembles the sensor network in the computer numerical control machine of Figure 1 is shown in Figure 4. Following the recommendations from [40,55], we build the quantum digital twin with a number of qubits equal to the number of sensors and one *ancilla* qubit that serves to perform the appropriate rotations, giving a total of 6 qubits in this case. In addition, as we have previously indicated in [41], after the proper qubit initialization, we perform a series of qubit rotation operations that allow us to simulate the conditional probabilities between the respective sensors. The interested reader can inspect several examples for several qubit configurations in these application examples from [42,43,44].

The factory in which this project was carried out has a workforce with a low level of education, which is why management has to look for intuitive solutions to visualize the state of the value creation processes so that workers can understand them and act on them. To this end, a visualization based on the logic of traffic lights, known to all workers, is used: green and red mean that the machine is operating within or outside specifications, respectively, while yellow shows a situation of caution as the machine is operating at the limit of specifications. The display of the state of the last qubit is performed by means of a sense HAT that presents a linear colour degradation given by the expression $RGB255\cdot [{∥\langle 1|q_{i}\rangle ∥}^{2},{∥\langle 0|q_{i}\rangle ∥}^{2}),0]$, i=0,…,5. This yields naturally to a green colour RGB[0,255,0] if the probability of the last qubit of the circuit $P\left(q_{5}=|0\rangle \right)={∥\langle 0|q_{5}\rangle ∥}^{2}=1$ and a red colour RGB[255,0,0] if the $P\left(q_{5}=|1\rangle \right)={∥\langle 1|q_{5}\rangle ∥}^{2}=1$ which is the standard traffic-light colour code in the shopfloor: green, yellow, and red.

## 3. Results

**Figure 5:**
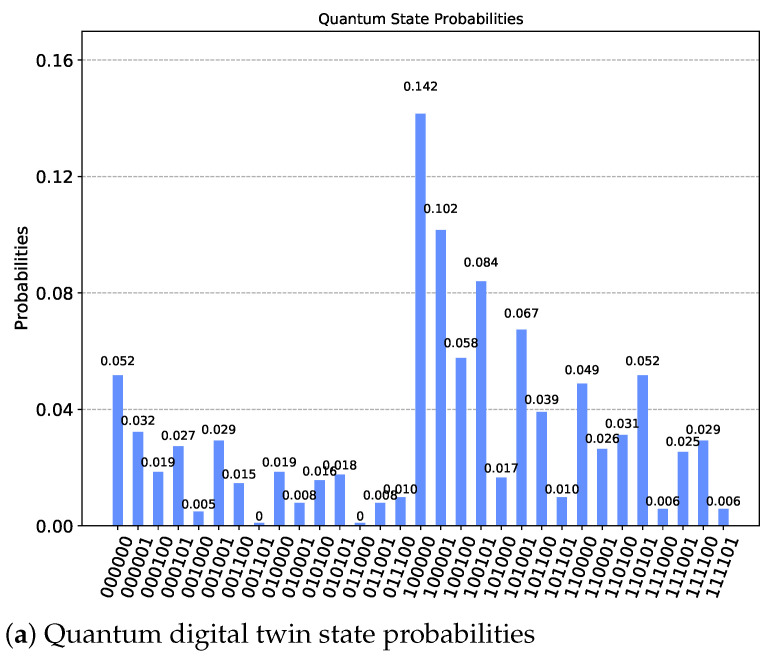

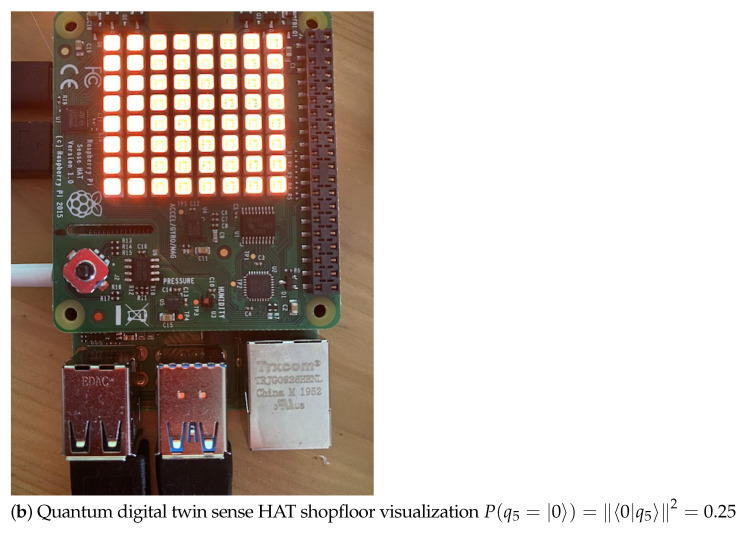
Quantum digital twin state probabilities and shopfloor visualization.

The results obtained by the simulation of the digital quantum twin can be represented in the form of a bar chart representing the quantum state probabilities of the quantum circuit, as shown in Figure 5a. However, this visualization is not intuitive and, therefore, has little chance of being interpreted satisfactorily by the process owner in an Industry 4.0 environment. Without this visual interpretation of the machine status, it is not possible to successfully perform a proper shopfloor management. For this reason, we have added an 8 × 8 RGB LED matrix display that allows a quick and intuitive visualization of the total state of the wave equation of the quantum circuit. This is exemplary shown in Figure 5b, which represent the sum of the 32 qubit combinations $P(q_{5}=|0\rangle )$ given by $P\left(q_{5}=|0\rangle \right)={∥\langle 0|q_{5}\rangle ∥}^{2}=0.25$, hence delivering a reddish visualization equivalent to RGB[191,64,0].

**Figure 6:**
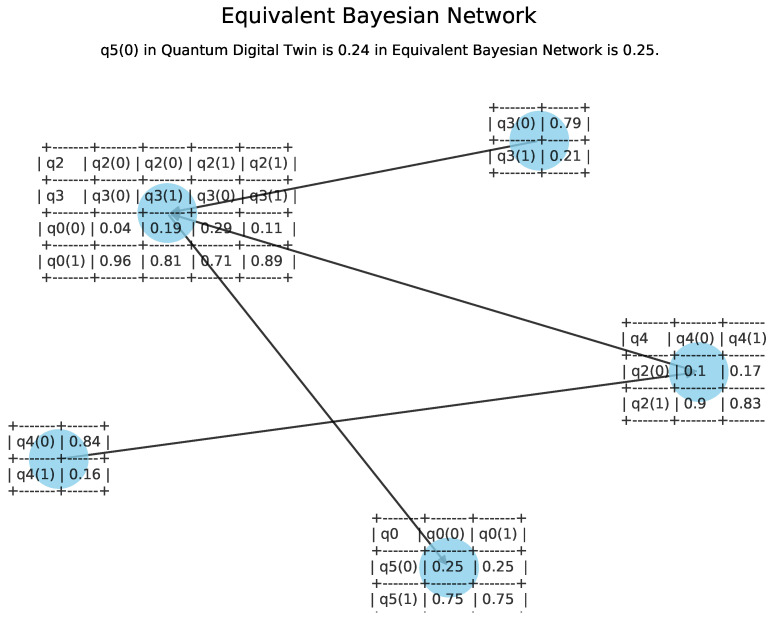
Equivalent Bayesian network to the quantum digital twin shown in Figure 4.

To verify that the result is correct, we proceed to design the equivalent Bayesian network to the digital quantum twin [40] represented in Figure 6. The error percentage found when comparing both quantum digital twin and the classical Bayesian network is less than 2%, which is acceptable in the context of quantum simulations. The computation time of the equivalent Bayesian network doubles the quantum digital twin computational performance, which is a pre-requisite for the implementation to be carried out in real time.

## 4. Discussion and Conclusions

In the initial situation, before starting the project, manual quality control was performed on 1% of the parts. The initial proposal of the process owner was to perform a condition monitoring of each of the sensors and aggregate this information by means of a digital twin with machine learning methods, which would reduce the cost of personnel associated with quality control. However, it would be difficult to integrate this into the production process due to two reasons: on the one hand, the lack of knowledge of these methods by the operators, and, on the other hand, the computational cost of performing calculations by classical methods (Bayesian networks) that do not allow integration in the production. Our quantum digital twin allows to obtain the two advantages: to obtain the information on the product quality in the form of an intuitive visualization for the operator in real time and at low cost. The integration of the quantum digital twin has meant a reduction in the costs associated with quality control, as well as doubling the mean-time-between-failures associated with the CNC machine as the speed of reaction of the operator in case of error is increased.

In this work, we have successfully tested the integration of a digital quantum twin by means of quantum simulations on a conventional machine to enable a visualization of its systemic state in an Industry 4.0 environment. With the case study, we have achieved the much desired integration of quantum computing logic in industrial environments and opened a field of exploration that will allow, once emulated, managers of value creation processes to use these algorithms in a clean and simple way. Digital quantum twins is much more than just software for reaching the same goal in a slightly better way. Instead, it does not develop in isolation, but the socio-technical system enables the development, diffusion and use of technologies. Digital quantum twins necessarily change processes and the way in which people work. With this work, we have generated a bridge, so far non-existent in practice, between the world of quantum simulation and industrial environments. We have also confirmed the validity of the results by demonstrating that the quantum digital twin yields the same values as those obtained with traditional simulation methods, such as Bayesian networks.

We have integrated a quantum simulation that allows on the one hand to monitor the state of a sensor network inside the machine and on the other, through an intuitive traffic light visualization, shopfloor management system, to empower the process owner to benefit from the quantum digital twin results without any quantum knowledge. This means that our proposal has the potential to be widely applied in practice since it requires neither a high investment, nor a redesign of its components, nor a specific knowledge of quantum simulation principles. From an autonomation view, these characteristics of usability, selective provision of information, user acceptance, and profitability could result not only in better human-machine cooperation systems, but also may lead to changes in the range, depth, and content of tasks.

The objective of our work is the practical application of quantum simulation in a real environment. For this purpose, we present a real implementation of a prototype connected to a CNC machine in industrial use. Nevertheless, the proposal has some limitations. Quantum computers constitute a huge investment due to the required physical functioning conditions. Currently, quantum computing is available either using external free resources as IBM *qiskit*, the one used in the prototype that has a limit of 30-qubits, or through rental of proprietary equipment. Escalation to an extensive industrial deployment is neither expected to imply relevant barriers from the conceptual and technological point of view, nor it affects the design and logic of the prototype. Should the company hire a more powerful quantum computer, it would not affect the prototype functioning. The extra qubits would allow to compute Bayesian networks composed of a higher number of sensors. Computational benefits would then be significantly higher as the increase rate of quantum computation times with the number of network nodes is much slower than binary logic computers. However, challenges remain in structuring the condition monitoring offer, due to the different domains of application, the characteristics of the existing information, and the final goal of the monitoring activities.

## Data Availability

The code and additional results can be accessed in this Open Access Repository: https://osf.io/24jrm/?view_only=a10d2e001e114807854b994616f8d4cf.

## Abbreviations

The following abbreviations are used in this manuscript:

CNC	Computer numerical control
CPS	Cyber-physical systems
I${}^{2}$C	Inter-integrated circuit
JIT	Just-in-time
LAN	Local area network
QC	Quantum computing
LED	Light emitting diode
RFID	Radio frequency identification
RGB	Red–Green–Blue
SFM	Shopfloor management
SMED	Single minute exchange of die
SPI	Serial peripheral interface
TPM	Total productive maintenance
TQM	Total quality management
UART	Universal asynchronous receiver/transmitter
